# Early-Remitting Neonatal Diabetes in a Growth-Restricted Preterm Infant Requiring Insulin: A Case Report

**DOI:** 10.7759/cureus.106025

**Published:** 2026-03-28

**Authors:** Abdelhakim Boulfouyoul, Kaoutar Ettoini, Abdallah Oulmaati

**Affiliations:** 1 Department of Pediatrics and Neonatology, Mohammed VI University Hospital Center, Tangier, MAR

**Keywords:** case report, hyperglycemia, insulin therapy, intrauterine growth restriction, neonatal diabetes mellitus, prematurity, transient neonatal diabetes mellitus

## Abstract

Neonatal diabetes mellitus is a rare cause of persistent hyperglycemia in early infancy and is usually monogenic. We describe a male preterm neonate born at 34 weeks’ gestation with intrauterine growth restriction who developed severe hyperglycemia from the first days of life. Plasma glucose reached a maximum of 570 mg/dL with persistent glycosuria, negative urine ketones, and a normal acid-base profile. C-peptide was profoundly suppressed (0.01 ng/mL), supporting marked endogenous insulin deficiency. Following optimization of glucose administration, intravenous short-acting regular human insulin was initiated with hourly glucose monitoring and complicated by early asymptomatic hypoglycemia requiring rapid correction and insulin dose reduction. As enteral feeds started, treatment was switched to micro-dosed subcutaneous short-acting regular human insulin with the use of a freshly prepared dilution and a pre-feed threshold strategy. Insulin requirements progressively decreased, and therapy was discontinued by three months of age with maintenance of normoglycemia and normal glycated hemoglobin (HbA1c). Growth and neurodevelopment were normal through two years of follow-up. This case highlights the importance of keeping neonatal diabetes in mind for preterm, growth-restricted infants with persistent severe hyperglycemia requiring insulin after exclusion of secondary causes, and also to master the details of practical safety considerations for insulin use in early life.

## Introduction

Neonatal diabetes mellitus (NDM) is a rare cause of persistent hyperglycemia presenting within the first six months of life and is most commonly of monogenic origin. In contrast to autoimmune type 1 diabetes, which rarely presents in this age group [1,2], transient neonatal diabetes is most commonly associated with 6q24 abnormalities and mutations in KCNJ11 or ABCC8 [2]. Other monogenic causes of neonatal diabetes, including INS variants, have also been described [3]. Early recognition is important since identifying the etiology guides prognosis, follow-up, and treatment in some genetic subtypes [3]. In affected newborns with prematurity or growth restriction, distinguishing NDM from secondary hyperglycemia resulting from physiologic immaturity or acute illness, medications, nutrition, or excessive glucose infusion can be difficult and may postpone the appropriate assessment [1,4]. We describe a growth-restricted preterm infant with severe insulin deficiency requiring insulin therapy and subsequent early remission, highlighting both the diagnostic challenge and the practical use of a cautious insulin micro-dosing strategy.

## Case presentation

Patient information

A male neonate born at 34 weeks’ gestation by cesarean section for fetal distress (severe bradycardia) was transferred to our neonatal unit on day of life (DOL) 4 for evaluation and management of severe hyperglycemia. Birth weight was 1680 g, consistent with small-for-gestational-age status. Apgar scores were not documented in the referral record [5]. Maternal and pregnancy history was unremarkable; there was no diabetes in the index pregnancy, no exposure to antenatal corticosteroids, and no maternal fever, clinical chorioamnionitis, prolonged rupture of membranes, or preeclampsia. There was no reported consanguinity and no family history of diabetes.

Clinical findings

The infant was first admitted to the referring hospital because of transient respiratory distress. During monitoring, capillary glucose values were repeatedly elevated (210-370 mg/dL) and reached 534 mg/dL on the day of transfer.

On admission to our unit (DOL 4), weight was 1700 g (3rd percentile), length 44 cm (50th percentile), and head circumference 33 cm (90th percentile) [6], consistent with asymmetric intrauterine growth restriction. Capillary glucose on admission was 397 mg/dL. On admission, temperature was 37.1°C, heart rate 120 beats/min, respiratory rate 40 breaths/min, and oxygen saturation 98% on room air, without respiratory distress (Silverman-Andersen score 0/10) [7]. He was conscious and reactive, with poor feeding and hypotonia, without apnea or seizures. There were no clinical signs of dehydration, and urine output was 2.8 mL/kg/h. No dysmorphic features were noted; macroglossia and umbilical hernia were absent.

Diagnostic assessment

Hyperglycemia was confirmed, and urinalysis consistently showed glycosuria with negative urine ketones. Plasma glucose peaked at 570 mg/dL on DOL 5. Acid-base evaluation showed a pH of 7.4 and a bicarbonate of 21 mmol/L.

Significant hyperglycemia was recorded multiple times from DOL 1-3 at the referring hospital and persisted after transfer (DOL 4-6), requiring insulin therapy. The infant was started on intravenous 10% dextrose in water (D10W) without parenteral nutrition in the initial days, and the estimated glucose infusion rate (GIR) was approximately 5.6-8.3 mg/kg/min. After transfer, the GIR reached approximately 9.0 mg/kg/min on DOL 6. There was no previous exposure to systemic corticosteroids, catecholamines/vasopressors, caffeine, or parenteral nutrition.

The infant remained clinically stable, and his sepsis evaluation was reassuring (complete blood count within the reference range, C-reactive protein (CRP) 2.9 mg/L, and blood and urine cultures negative).

Initial metabolic tests revealed sodium 138.6 mmol/L, potassium 4.99 mmol/L, chloride 104.4 mmol/L, calcium 9.3 mg/dL, urea 46 mg/dL, and creatinine 0.7 mg/dL. C-peptide was 0.01 ng/mL (reference range 1.1-4.4 ng/mL), measured on DOL 6 after insulin had already been initiated; concomitant glucose at the time of sampling was approximately 500 mg/dL.

A cranial ultrasound was performed, given hypotonia and poor feeding on admission, which was normal. Abdominal ultrasound showed bilateral hydronephrosis without a visible obstructive lesion. Pancreatic magnetic resonance imaging (MRI) was performed, but was non-diagnostic due to motion artifact.

Therapeutic intervention

Before transfer, the infant received intravenous D10W with electrolytes as maintenance fluids. The dextrose volume was advanced from approximately 80 mL/kg/day on DOL 1 and increased by approximately 10 mL/kg/day, reaching approximately 120 mL/kg/day by DOL 5 (estimated GIR approximately 5.6-8.3 mg/kg/min).

On DOL 4, intravenous short-acting regular human insulin produced by recombinant DNA technology [8] was initiated via an electric syringe pump at 0.05 units/kg/h with hourly capillary glucose monitoring. Early asymptomatic hypoglycemia occurred (nadir 20 mg/dL, confirmed on plasma testing) and was managed by stopping the infusion and administering an intravenous D10W bolus (2 mL/kg), followed by repeat glucose assessment. When hyperglycemia recurred, the infusion was resumed at 0.02 units/kg/h. Insulin was held for any capillary glucose <45 mg/dL; this conservative approach was chosen to minimize the risk of hypoglycemia in a small preterm infant.

With established breastfeeding and persistent insulin requirement, treatment was transitioned on DOL 6 to subcutaneous short-acting regular human insulin using a pre-feed threshold strategy. A conservative weight-based micro-dosing plan was used: 0.1-0.15 units/kg/dose every six hours before feeds, administered only if pre-feed capillary glucose was >200 mg/dL. A pre-feed threshold of >200 mg/dL was used to target persistent clinically significant hyperglycemia while maintaining a cautious safety margin in this high-risk preterm infant [4]. For example, at 1.7 kg, 0.12 units/kg is approximately 0.2 units. Insulin pens were not used because the minimum deliverable dose exceeded the required microdose; subcutaneous doses were therefore administered by syringe using a freshly prepared dilution under a standardized protocol with trained nursing administration and structured caregiver education.

For micro-dosing, U-100 insulin was diluted with normal saline to 10 units/mL by mixing 0.1 mL (10 units) of U-100 insulin with 0.9 mL normal saline (final volume 1.0 mL). The dilution was prepared fresh for each dose and not stored. With this dilution, 0.01 mL corresponds to 0.1 units; therefore, 0.2 units equals 0.02 mL, which corresponds to the two-unit mark on a U-100 insulin syringe.

Follow-up and outcomes

Insulin was tapered as capillary glucose values improved. Intermittent pre-feed hyperglycemia persisted after intravenous dextrose was discontinued and during exclusive breastfeeding, requiring the conditional subcutaneous insulin plan until discharge (DOL 35).

Before discharge, the mother completed structured, competency-based training, including supervised dilution preparation, dose measurement, injection technique, pre-feed glucose checks, and recognition and management of hypoglycemia. She was held until she could consistently perform the correct preparation and administration while under nursing observation. At early follow-up visits, technique and home glucose logs were reviewed, and safety steps were reinforced.

The infant was discharged on DOL 35 with conditional subcutaneous insulin (0.1-0.15 units/kg/dose every six hours only if pre-feed capillary glucose was >200 mg/dL) and home capillary glucose monitoring before feeds (four to six times/day). Follow-up was scheduled at 48 hours, then twice weekly for two weeks, weekly for one month, every two weeks afterwards, and finally monthly.

Insulin was discontinued by three months of age with sustained normoglycemia (typically 90-110 mg/dL). At three months, glycated hemoglobin (HbA1c) was 5.5%, and glutamic acid decarboxylase (GAD) and insulinoma-associated antigen-2 (IA-2) antibodies were negative. Follow-up renal ultrasound showed spontaneous resolution of bilateral hydronephrosis. Repeat pancreatic MRI was not pursued after remission. At two years of follow-up, growth trajectories were appropriate for corrected age, and neurodevelopmental examinations were normal, without relapse.

The clinical course is summarized in Table 1.

**Table 1:** Clinical timeline Estimated GIR for D10W was calculated as (mL/kg/day × 100 mg/mL)/1440; for example, 120 mL/kg/day corresponds to 8.3 mg/kg/min. DOL: day of life; D10W: 10% dextrose in water; GIR: glucose infusion rate; IV: intravenous; SC: subcutaneous; HbA1c: glycated hemoglobin

Day of life/age	Nutrition/IV fluids	Estimated GIR (mg/kg/min)	Glucose (mg/dL)	Insulin (route + dose)		Key events	
DOL 1-3 (referring hospital)	D10W 80-100 mL/kg/day	5.6-6.9	210-370	None		Hyperglycemia was documented during monitoring prior to transfer	
DOL 4 (our unit)	D10W 110 mL/kg/day	7.6	397 on admission; up to 534	IV regular insulin 0.05 U/kg/h		Transfer; IV insulin initiated; hourly glucose monitoring	
DOL 5 (our unit)	D10W 120 mL/kg/day	8.3	Peak 570	IV regular insulin 0.02 U/kg/h		Asymptomatic hypoglycemia nadir 20 mg/dL; insulin held; IV D10W bolus 2 mL/kg; dose reduced	
DOL 6 (our unit)	D10W 130 mL/kg/day	9.0	Recurrent hyperglycemia	Transition to SC insulin; conditional plan (0.1-0.15 U/kg/dose every six hours if pre-feed >200 mg/dL)		Enteral feeds established; switch to SC regimen; C-peptide 0.01 ng/mL	
DOL 7-35 (our unit)	Weaned off IV D10W; breastfeeding established	-	52-290	SC conditional plan continued; gradual taper		Intermittent pre-feed hyperglycemia persisted; caregiver training and supervision	
DOL 35 (discharge)	Breastfeeding only	-	~100-200	SC conditional plan continued		Discharge with structured education and a home monitoring plan	
Three months	Breastfeeding only	-	Typically 90-110	Stopped		Insulin discontinued; HbA1c 5.5%	
Two years	Normal diet for age with continued breastfeeding	-	Typically 90-110	None		No relapse; growth and neurodevelopment are normal	

## Discussion

NDM is a rare form of diabetes that presents during the first six months of life. It should be differentiated from transient forms of hyperglycemia commonly observed in premature infants in neonatal intensive care units [1,2]. NDM is traditionally divided into transient neonatal diabetes mellitus (TNDM) and permanent neonatal diabetes mellitus (PNDM) [1,2]. TNDM usually remits in early life, typically within the first three to six months of age, whereas PNDM requires lifelong therapy [9,10].

In preterm infants, hyperglycemia is often secondary to physiologic immaturity and stressors such as infection, medications including corticosteroids, catecholamines, and caffeine, parenteral nutrition, or excessive glucose infusion, and it often improves after stabilization and optimization of glucose delivery [4]. In this infant, marked hyperglycemia was reported repeatedly from DOL 1 and continued after breastfeeding, with episodic hyperglycemia before feeds through discharge (DOL 35), despite clinical stability, reassuring blood cultures and inflammatory markers, absence of relevant medication exposures, and avoidance of parenteral nutrition [1,2,4]. Parenteral nutrition was not administered, and the GIR remained within the normal neonatal range, but severe hyperglycemia persisted, raising a strong suspicion of a primary insulin-deficient process. Persistent glycosuria in the absence of ketosis or acidosis and severely suppressed C-peptide during marked hyperglycemia also argued against isolated hyperglycemia of prematurity and favored a primary insulin-deficient process [1,2]. Although obtained after insulin initiation, C-peptide reflects endogenous secretion and is absent from exogenous insulin. Therefore, a markedly suppressed value in the setting of severe hyperglycemia supports profound insulin deficiency [1,2]. At sampling, glucose was approximately 500 mg/dL, a level that would normally stimulate endogenous insulin secretion.

Current guidelines recommend genetic testing when diabetes is diagnosed before six months of age to confirm etiology, guide prognosis, including relapse risk, and potentially change therapy [3]. When available, testing typically includes evaluation for 6q24 abnormalities and sequencing of common monogenic diabetes genes such as KCNJ11, ABCC8, and INS. Identification of a K-ATP channel mutation may permit transition from insulin to an oral sulfonylurea in selected patients [11]. Genetic testing was desired to confirm the etiology and to counsel the family about relapse risk, but it was not available in our setting during evaluation, so molecular confirmation could not be made. The clinical course, with severe insulin deficiency requiring insulin and remission by three months, was compatible with a transient neonatal diabetes phenotype with early remission [9,10].

Management aims to treat hyperglycemia while minimizing iatrogenic hypoglycemia, which is particularly dangerous in small preterm infants [1,4]. Practical steps include optimizing GIRs and addressing reversible causes before initiating insulin [4]. When insulin is required, continuous intravenous infusion allows fine titration; published approaches include infusion rates as low as 0.02 units/kg/h, with many very low birth weight protocols starting near 0.05 units/kg/h and monitoring glucose hourly at the beginning [4]. In our case, early hypoglycemia highlighted the narrow therapeutic window in small preterm infants and the need for stepwise titration and clear hypoglycemia management. Commonly recommended strategies include adjusting the dose in small increments, for example, 0.01 units/kg/h, and avoiding rapid declines in glucose [4].

Once enteral feeds are established, transition to subcutaneous insulin is recommended if hyperglycemia persists [4]. Subcutaneous insulin is generally administered when glucose values before feeds are clearly elevated, for example, above approximately 200-250 mg/dL, with conservative starting doses such as 0.1-0.15 units/kg/dose to reduce the risk of hypoglycemia [4]. While rapid-acting insulin analogs are often preferred for multiple daily injections, they may be unavailable in some settings. In our unit, human regular insulin was used with a conditional dosing rule, careful administration of very small doses using a fresh dilution, and structured caregiver education to reduce dosing error. In infants, insulin delivery limitations are clinically important: without dilution, the smallest practical injectable dose may exceed the required dose, and safe dosing may require dilution or delivery methods supported by technology [4]. Emerging insulin delivery technologies, including pumps and continuous glucose monitoring, and in selected cases automated or closed-loop approaches, may improve safety and glycemic stability in infants with neonatal diabetes, although implementation depends on availability and local expertise [12]. Because bedside dilution is off-label and prone to error, such approaches should be reserved for settings where caregivers can complete supervised training with close follow-up after discharge.

Long-term outcomes vary by genetic subtype. In transient neonatal diabetes phenotypes with early remission, prognosis is often favorable once blood glucose stabilizes, though relapse can occur later in childhood or adolescence [2,9,10]. In this case, insulin was discontinued after three months with sustained normoglycemia. In early infancy, HbA1c may be less reliable, including in infants under six months, because of high fetal hemoglobin, and alternative markers such as glycated albumin may better reflect glycemic control in neonatal diabetes [4,13]. Serial home glucose logs together with sustained normoglycemia supported remission.

Limitations include the absence of genetic testing, the measurement of C-peptide after insulin initiation rather than before treatment, and a non-diagnostic pancreatic MRI; therefore, the diagnosis remains phenotype-based rather than molecularly confirmed.

## Conclusions

This case highlights the importance of considering neonatal diabetes in preterm, growth-restricted infants with severe persistent hyperglycemia after exclusion of more common secondary causes. It also emphasizes the narrow therapeutic window of insulin therapy in this setting and the value of practical safety measures, including careful micro-dosing, hypoglycemia management, and structured caregiver education, to support safe treatment until remission.
